# KLF8 overexpression promotes the growth of human lung cancer cells by promoting the expression of JMJD2A

**DOI:** 10.1186/s12935-019-0970-3

**Published:** 2019-10-07

**Authors:** Dongjie Ma, Hongsheng Liu, Yingzhi Qin, Zhenhuan Tian, Shanqing Li, Naixin Liang

**Affiliations:** Department of Thoracic Surgery, Peking Union Medical College Hospital, Chinese Academy of Medical Sciences & Peking Union Medical College, Beijing, 100730 China

**Keywords:** Lung cancer, KLF8, Cell cycle, JMJD2A, P21, CDK4

## Abstract

**Background:**

Non-small-cell lung cancer (lung cancer) has become one of the leading causes worldwide and the underlying mechanism is not fully understood. The transcriptional factor Kruppel like factor 8 (KLF8) is involved in the initiation, progression, transformation, and metastasis of diverse cancers. However, the roles of KLF8 in human non-small cell lung cancer remain unknown.

**Methods:**

CCK-8 kit and colony formation assay were performed to determine the cell growth of lung cancer cells. Flow cytometry analysis was used to evaluate apoptosis and cell cycle of lung cancer cells. Luciferase reporter assay was used to examine the activation of JMJD2A promoter by KLF8. Chromatin immunoprecipitation assay was performed to evaluate the binding of KLF8 to JMJD2A promoter. Western blot and polymerase chain reaction were applied to analyze the expression of interested genes.

**Results:**

The mRNA and protein levels of KLF8 in human non-small cell lung cancer tissues were overexpressed compared with the non-cancer tissues. KLF8 was knocked down with lentivirus-mediated short-hairpin RNA (shRNA) in human lung cancer cells (A549 and H1299 cells). The phenotypic results showed that KLF8 knockdown decreased the proliferation rate and colony formation of lung cancer cells. By contrast, lentivirus-mediated KLF8 overexpression promoted the growth of lung cancer cells (A549 and H1299 cells) and non-cancerous bronchial epithelial cell line BEAS-2B. Next, we showed that KLF8 regulated cell cycle at the G0 phase but not regulates cellular apoptosis of lung cancer cells. KLF8 regulated the expression of the cell cycle regulators P21 and CDK4 in a JMJD2A-dependent manner and JMJD2A knockdown significantly blocked the functions of KLF8 in regulating cell cycle and proliferation of lung cancer cells. Finally, we observed that KLF8 bound the promoter of JMJD2A and facilitated the expression of JMJD2A.

**Conclusions:**

Our evidence demonstrated that KLF8 upregulation in human lung cancer promotes the cell proliferation and colony formation of lung cancer cells. KLF8 binds to the promoter of JMJD2A and subsequently regulates the expression of P21 and CDK4, which contributes to the regulation of cell cycle by KLF8. KLF8 may serve as a target for the treatment of human lung cancer.

## Background

Of the deaths from cancer, non-small-cell lung cancer (lung cancer) has become one of the leading causes worldwide. The improvements in understanding of the mechanism underlying this disease are urgently needed for better diagnostics and treatment application [1]. During the past decade, single-cell based transcriptional and genomic analysis, the in-depth analyses of lung cancer genomes, and exploring of core signaling pathways have further defined lung cancers as a group of distinct diseases with genetic and cellular heterogeneity [2]. Despite these intensive efforts to comate defeat lung cancer, the prognosis of this disease remains unfavorable and is especially miserable poor in advanced lung cancer [3]. Therefore, it is still very important to have a better understanding of the molecule-based underlying mechanism.

Kruppel-like factor 8 (KLF8) is one of the members of the family of KLF transcription factor family. KLF8 contains a conserved DNA-binding zinc-finger domain on its C-terminus and the N-terminal that determines its functional specificity [4]. KLF8 acts as a target of focal adhesion kinase (FAK) in cell cycle regulation [5]. KLF8 is critically involved in v-Src-induced transformation and plays a critical role in tumor progression [6]. In human breast cancer, KLF8 promotes the invasion and metastasis of cancer cells by promoting the expression of matrix metalloproteinase 9 (MMP9) [7]. Furthermore, KLF8 promotes tumorigenesis, invasion, and metastasis of colorectal cancer cells by activating four and a half LIM protein 2 (FHL2). Additionally, *KLF8* knockdown triggers growth inhibition and induces arrest of the cell cycle in human pancreatic cancer cells [8]. However, the roles of KLF8 in human lung cancer remains unknown.

JMJD2A is a histone demethylase that participates in diverse aspects of physiological and pathological progress. The roles of JMJD2A in regulating cancer biology are also identified [9]. For instance, JMJD2A shows oncogenic feathers in human breast cancers [10]. JMJD2A contributes to breast cancer progression through repressing the expression of the tumor suppressor Aplasia Ras homolog member I (ARHI) [11]. Through repression of the tumor suppressor chromodomain-helicase DNA binding protein 5 (CHD5), JMJD2A blocks cellular senescence and promotes cellular transformation [12]. JMJD2A is remarkably overexpressed in human lung cancer and regulates the cell cycle of lung cancer cells and a high level of JMJD2A predicts a poor prognosis in patients with lung cancer [12–15]. Furthermore, JMJD2A protein level is upregulated in a cell cycle-dependent manner. JMJD2A overexpression increases chromatin accessibility, altered replication timing of specific genomic loci and leading the S phase progression [16]. In addition, depletion of JMJD2A leads to cell cycle arrest and subsequently p53-dependent senescence [12]. JMJD2A deregulation is critically in human carcinogenesis via regulating the G1/S transition [13].

Here in the present report, we demonstrate that KLF8 overexpression in human lung cancer promotes cell cycle progress via a JMJD2A-dependent manner. We observed that the expression levels of KLF8 were overexpressed in human lung cancer tissues and KLF8 facilitated the proliferation and colony formation of human lung cancer cells. KLF8 regulated the cell cycle but not survival of lung cancer cells depending on its regulation of the expression of the histone demethylase JMJD2A.

## Materials and methods

### Human lung cancer tissues

We collected lung cancer tissues (n = 34) and adjacent non-cancer lung tissues (n = 16) at Peking Union Medical College Hospital from 2011–2018 (Table 1). The collected tissue samples were transferred to − 80 °C immediately before RNA and protein extraction. This study was approved by the Ethics Committee for the patients-based study of the Peking Union Medical College Hospital. The written informed consent was obtained from each patient.

**Table 1:** Baseline characteristics of 34 patients with lung cancer

Characteristic	N (%)	KLF8 low (n = 17)	KLF8 high (n = 17)	p value
Age				
> 60	19 (56%)	9	10	>0.999
≤ 60	15 (44%)	8	7	
Gender				
Male	21 (62%)	10	11	>0.999
Female	13 (38%)	7	6	
Smoking history				
Ever smoker	22 (65%)	10	12	0.721
Never smoker	12 (35%)	7	5	
TNM stage				
I/II	15 (44%)	4	11	0.016
III/IV	19 (56%)	13	6	
Histological subtype				
Adenocarcinoma	18 (53%)	10	8	0.732
Squamous cell carcinoma	16 (47%)	7	9	
Lymph node metastasis				
Yes	11 (32%)	2	9	0.026
No	23 (68%)	15	8	
Tumor differentiation				
Poor	14 (41%)	7	7	0.940
Moderate	15 (44%)	8	7	
Well	3 (9%)	1	2	
None	2 (6%)	1	1	
EGFR mutation				
Yes	7 (21%)	3	4	0.671
No	27 (79%)	14	13	

### Immunohistochemical analysis

The immunohistochemical experiment was performed based on the method described in previous work [17]. Briefly, the tumor and control lung tissues were fixed in 4% paraformaldehyde (Servicebio) and then coated in paraffin. Then, the samples were sectioned into 5-µm-thick slices. For the immunohistochemical experiment, the slides were first deparaffnized, followed by cancellation of endogenous peroxidase activity with 3% (v/v) hydrogen peroxide. Non-specific binding sites were blocked with 10% bovine serum (Beyotime) for 1 h at room temperature. The slides were incubated at 4 °C overnight with diluted anti-KLF8 antibody (Abcam,#ab168527) and then with a biotinylated secondary antibody (Beyotime) at 37 °C for 30 min before subsequent incubation with an HRP-conjected streptavidin solution (Beyotime) for 20 min at 37 °C. The protein content in cancer tissues was analyzed with Image Pro Plus software (Media Cybernetics).

### Cell lines and cell culture

Five cancer cell lines (A549, H1975, H1299, H460, and H520) and one non-cancerous bronchial epithelial cell line (BEAS-2B) were involved in this study. All of these cell lines were obtained from the American Type Culture Collection (ATCC). The cells were cultured in DMEM (Dulbecco’s Modified Eagle’s Medium) medium (ThermoFisher, #11875-093) supplemented with 10% fetal bovine serum (Hyclone, #16000-044), 100 units/ml penicillin and 100 µg/ml streptomycin (ThermoFisher, # 15140122). All cells were cultured at 37 °C under a humidified atmosphere containing 5% CO_2_.

### Lentivirus packaging and transduction

To overexpression or knockdown of KLF8 or JMJD2A, the lentivirus system was applied. The short hairpin RNAs (shRNA) targeting KLF8 or JMJD2A were annealed and cloned into pLKO1.1-puro. shKLF8, shJMJD2A, and control shRNA lentivirus particles were purchased from Invitrogen. The shRNA targeting KLF8 is as follow: shKLF8-1# 5′-GCCATTACAGTCCCACTCAT-3′, shKLF8-2# 5′-CCCAGCACTGTTTAATGACA-3′; and shRNA targeting *JMJD2A* is as follow: 5′-TTCGAGAGTTCCGCAAGATAG-3′. To overexpress human *KLF8*, the human *KLF8* open reading frame was cloned into a pLV105 plasmid. To produce lentivirus, HEK293T cells were co-transfected with the lentivirus particles with psPAX2 (Addgene, #12260) and pLV-VSVG (Addgene, #82724), the two plasmids express lentivirus background constructs. A549 and H1299 cells were infected with lentivirus in the presence of polybrene (8 mg/ml) for 48 h. For transduction, the infected A549, H1299, and BEAS-2B cells were selected with puromycin (2 ugs/ml) for an additional 72 h.

### Quantitative real-time PCR (qRT-PCR)

Fresh human lung cancer tissues or cultured cells were subjected to RNA extraction with TRIzol reagent (ThermoFisher, #15596026). Then, 1 ug of total RNA was subjected to the synthesis of the first strand of cDNA with the Advantage RT-for-PCR kit (Clontech, #PCR5914). Next, qRT-PCR was applied to analyze the mRNA level of target genes with SYBR Green II (Takara, #RR820). The following pairs of primers were used in this study:

*KLF8* forward: 5′-CCCAAGTGGAACCAGTTGACC-3′

*KLF8* reverse: 5′-GACGTGGACACCACAAGGG-3′

*JMJD2A* forward: 5′-ATCCCAGTGCTAGGATAATGACC-3′

*JMJD2A* reverse: 5′-ACTCTTTTGGAGGAACAACCTTG-3′

*GAPDH* forward: 5′-TGTGGGCATCAATGGATTTGG-3′

*GAPDH* reverse: 5′-ACACCATGTATTCCGGGTCAAT-3′

*P21* forward: 5′-TGTCCGTCAGAACCCATGC-3′

*P21* reverse: 5′-AAAGTCGAAGTTCCATCGCTC-3′

*CDK4* forward: 5′-ATGGCTACCTCTCGATATGAGC-3′

*CDK4* reverse 5′-CATTGGGGACTCTCACACTCT-3′

### Western blot

Hunan lung cancer tissues or cultured cells were extracted for protein with RIPA lysis buffer (Merck Millipore, #20-188) supplied with protease inhibitor cocktail (Sigma, #P8340). Then, 30 ugs of total protein were subjected to western blot with the standard protocol as described elsewhere [18]. The primary antibodies used in this study are listed as follow: anti-KLF8 antibody (Abcam, #ab168527), anti-JMJD2A antibody (Abcam, #ab105953), anti-P21 antibody (Cell Signaling Technology, #2947), anti-GAPDH antibody (Abcam, #ab9485). The secondary antibodies were purchased from ThermoFisher (#G-21040 and #G-21234). The Chemiluminescent ECL reagent was purchased from Beyotime (#P0018).

### Cell proliferation experiment

The A549 and H1299 cells were plated in 96-well plates. The proliferation rate of lung cancer cells was evaluated with Cell Counting Kit-8 (MedChemExpress, #HY-K0301) in according to the protocol at 24, 48 and 72 h post cell plating. The number of cells was normalized to the control group at day 0 (%) and the percentage of cell number was shown.

### Colony formation experiment

Transduced cells were subjected to analysis the capacity of colony formation with soft agar assay. For this experiment, the equal number of transduced cells were cultured with appropriate controls in a soft agar medium for 2 weeks. The clones were stained with Crystal Violet Cell Colony Staining Kit (GenMed, #GMS10007). The number of clones per well of 6-well plates were analyzed by Image J and the relative number of colonies was shown.

### Apoptosis analysis

The apoptosis of A549 cells was analyzed with the Annexin V-APC apoptosis detection kit (Ebioscience; #88-8007-72). The flow results were analyzed within the FlowJo software and the percentage of apoptotic cells was qualified.

### Cell cycle assay

Flow cytometry was applied to analyze the cell cycle. Briefly, A549 cells cultured in 6-well plates were in logarithmic phase for 96 h. Next, the cells were plated to a new 6-well plate. 48 h later, A549 cells were fixed in 70% cold ethanol at 4 °C overnight. Then, the cells were washed with ice-cold PBS for twice followed by staining with propidium iodide (PI) buffer containing 10 mg/ml RNase at 37 °C. Then cell fluorescence was analyzed with flow cytometry. The flow results were analyzed within the FlowJo software and the percentage of cell cycle phase (G0/G1, S, and G2/M) were qualified.

### Chromatin immunoprecipitation assay

Chromatin immunoprecipitation (ChIP) was performed with the ChIP-IT Express Enzymatic kit (Active Motif, #53009). In brief, chromatin from cultured A549 cells was cross-linked with 1% formaldehyde at 22 °C for 15 min. Then the chromatin was sheared to an average size of ~ 500 bp followed by immunoprecipitation with anti-KLF8 or anti-IgG antibodies. Finally, the ChIP-PCR was performed to analyze the binding of KLF8 to JMJD2A promoter. The primers for amplifying JMJD2A promoter was designed based on the KLF8 binding site.

### Luciferase-reporter analysis

The promoter of human JMJD2A (− 2500 bp to + 100 bp) was cloned into the pGL3 plasmid (Addgene, #64784) to generate pGL3-JMJD2A plasmid. A549 cells were infected with lenti-shKLF8, lenti-KLF8 or the control lentivirus and selected with puromycin to generate A549 cells with KLF8 overexpression of knockdown. Then, the related plasmids (pGL3-JMJD2A or pGL3-Ctrl) in combination with a pRL-TK plasmid (Promega, #E2231) were transfected in A549 cells for 24 h and luciferase assay was performed using a luciferase assay system (#E1500) from Promega.

### Statistical analysis

All data are shown as mean ± SEMs. Student’s *t* test was performed to analyze the difference between the two groups. For more than two groups, the one-way ANOVA analysis was performed to analyze the difference.

## Results

### The transcriptional factor KLF8 is increased in human lung cancer tissues

**Fig. 1:**
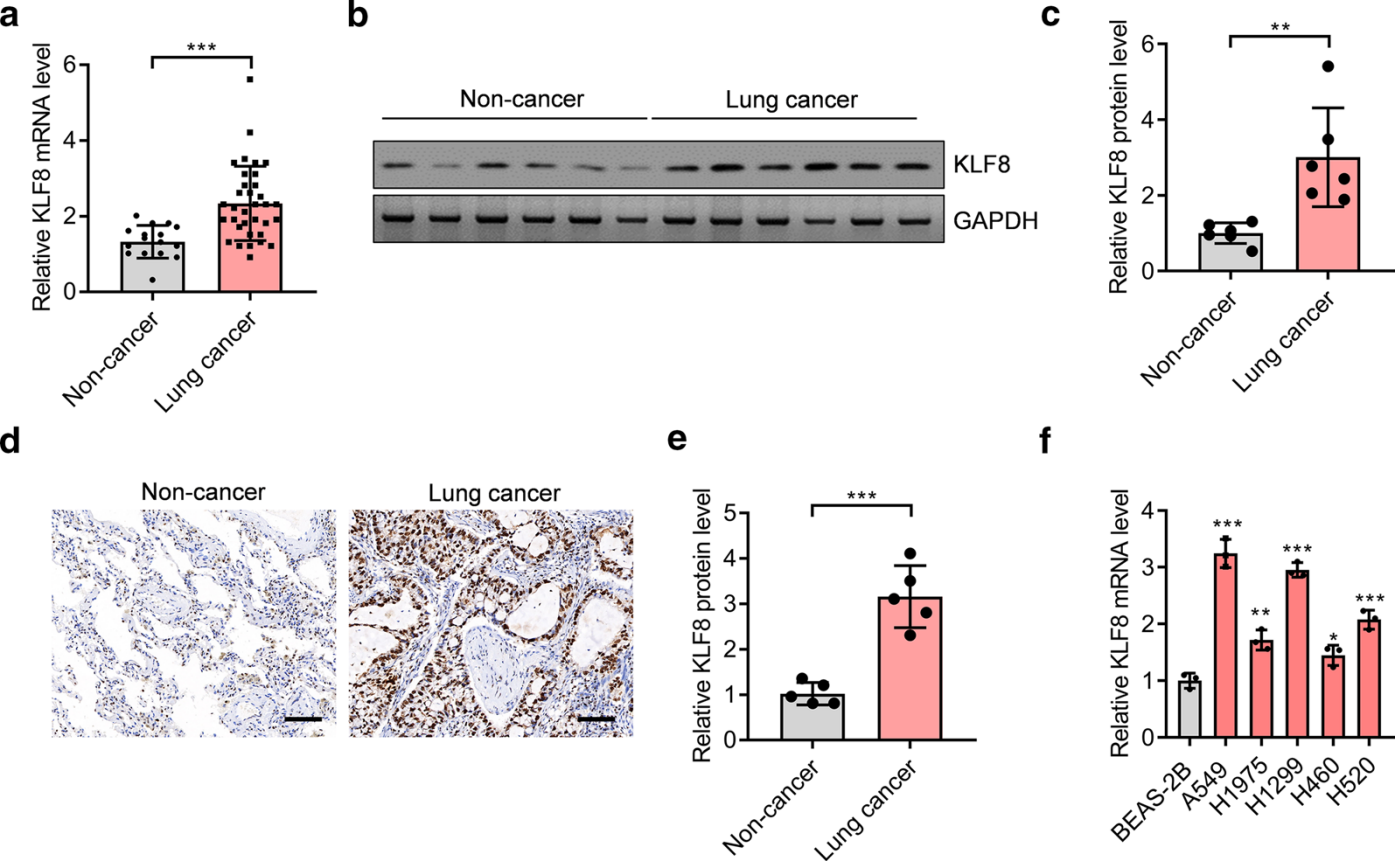
KLF8 is overexpressed in human lung cancer tissues. **a** The mRNA level of *KLF8* is overexpressed in human lung cancer tissues (n = 34) compared with non-cancer tissues (n = 16). ***p < 0.001. **b** Western blot results showing KLF8 expression in human lung cancer tissues and non-cancer tissues. **c** Quantifications of KLF8 protein level analyzed by western blot. N = 6, **p < 0.01. **d** Representative histochemical staining of KLF8 expression in non-cancer and lung cancer tissues. Bar = 100 μm. **e** Quantifications of KLF8 expression in non-cancer (n = 5) and lung cancer tissues (n = 5).***p < 0.001. **f** The mRNA level of *KLF8* is overexpressed in human lung cancer cell lines (A549, H1975, H1299, H460, and H520) compared with normal lung epithelial cell line (BEAS-2B). *p < 0.05, **p < 0.01 and ***p < 0.001. n = 3 in each group

To investigate the function of *KLF8* in human lung cancer, we first tested the expression of *KLF8* in human lung cancer tissues. To this end, we collected fresh lung cancer tissues (n = 34) and adjacent non-cancer tissues (n = 16), and then we analyzed the mRNA and protein levels of *KLF8* with qRT-PCR and western blot respectively. The results showed that the mRNA and protein levels of *KLF8* were significantly increased in human lung cancer tissues compared with adjacent non-cancer tissues (Fig. 1a–c). Our further immunohistochemical staining assay also confirmed that KLF8 expression was overexpressed in lung cancer tissues compared with non-cancer tissues (Fig. 1d, e). Importantly, we observed that high expression of KLF8 was associated with high TNM stage and metastasis, but not with cancer type (Table 1). In addition, KLF8 was highly expressed in human lung cancer cells lines compared with normal lung epithelial cells, and A549 and H1299 expressed the highest levels of KLF8 among the tested lung cancer cell lines (Fig. 1f). This finding implicates that KLF8 may play an important role in the development of human lung cancer.

### KLF8 promotes the proliferation and colony formation of lung cancer cells

**Fig. 2:**
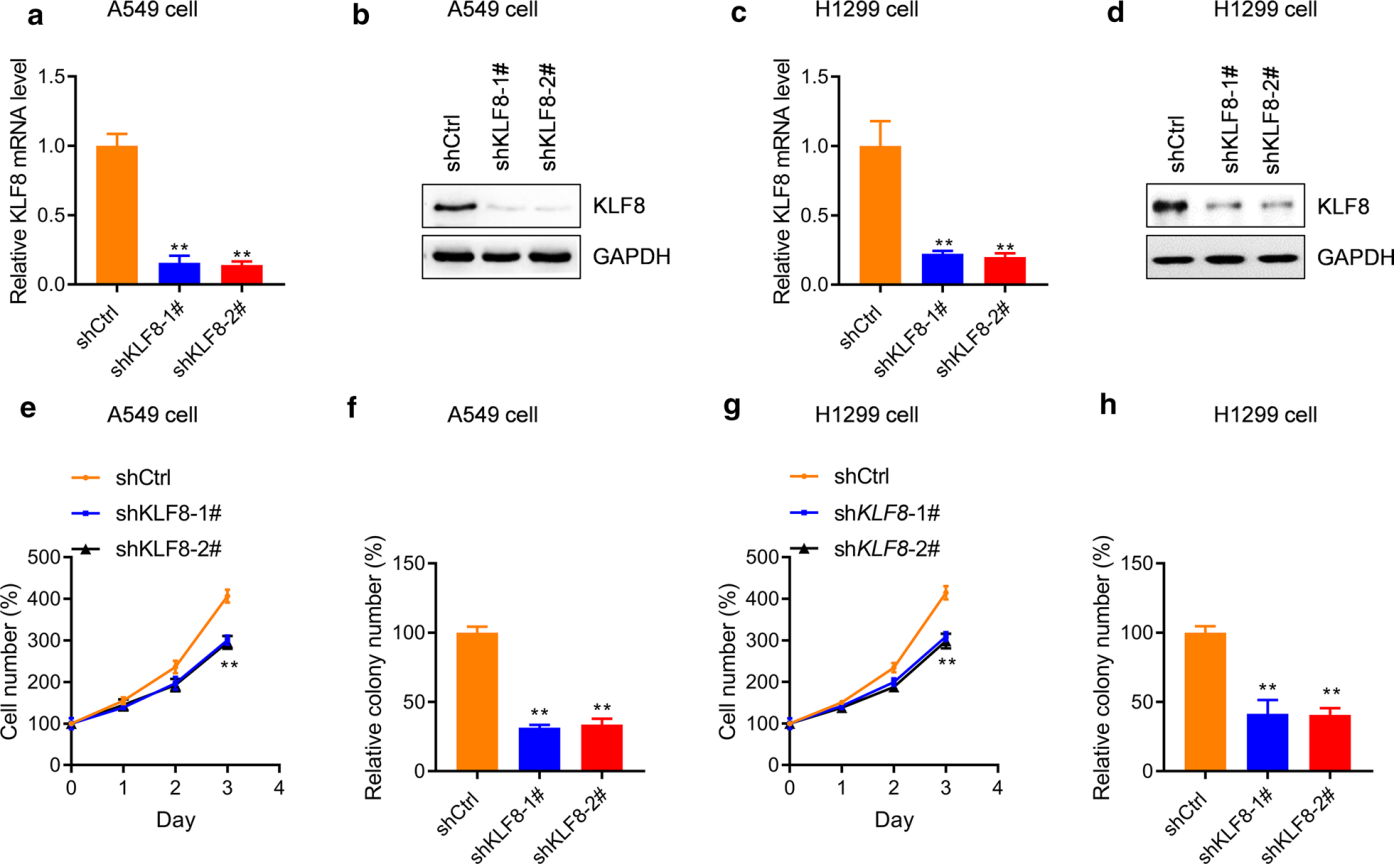
*KLF8* knockdown reduces the growth of lung cancer cells in vitro. **a**–**d** A549 and H1299 cells were infected with lentivirus carrying sh*KLF8* or control shRNA and were selected with puromycin. The transduced cells were subjected to analyze the mRNA (**a**, **c**) and protein (**b**, **d**) level of *KLF8*. **p < 0.01 *vs.* shCtrl. n = 3 in each group. **e**, **f** A549 and H1299 cells with/without sh*KLF8* transduction were subjected to proliferation assay (**e**, **g**) and colony formation assay (**f**, **h**). **p < 0.01 *vs.* shCtrl. n = 3 in each group. The number of cells was normalized to the control group at day 0 (%) and the percentage of cell number was shown

**Fig. 3:**
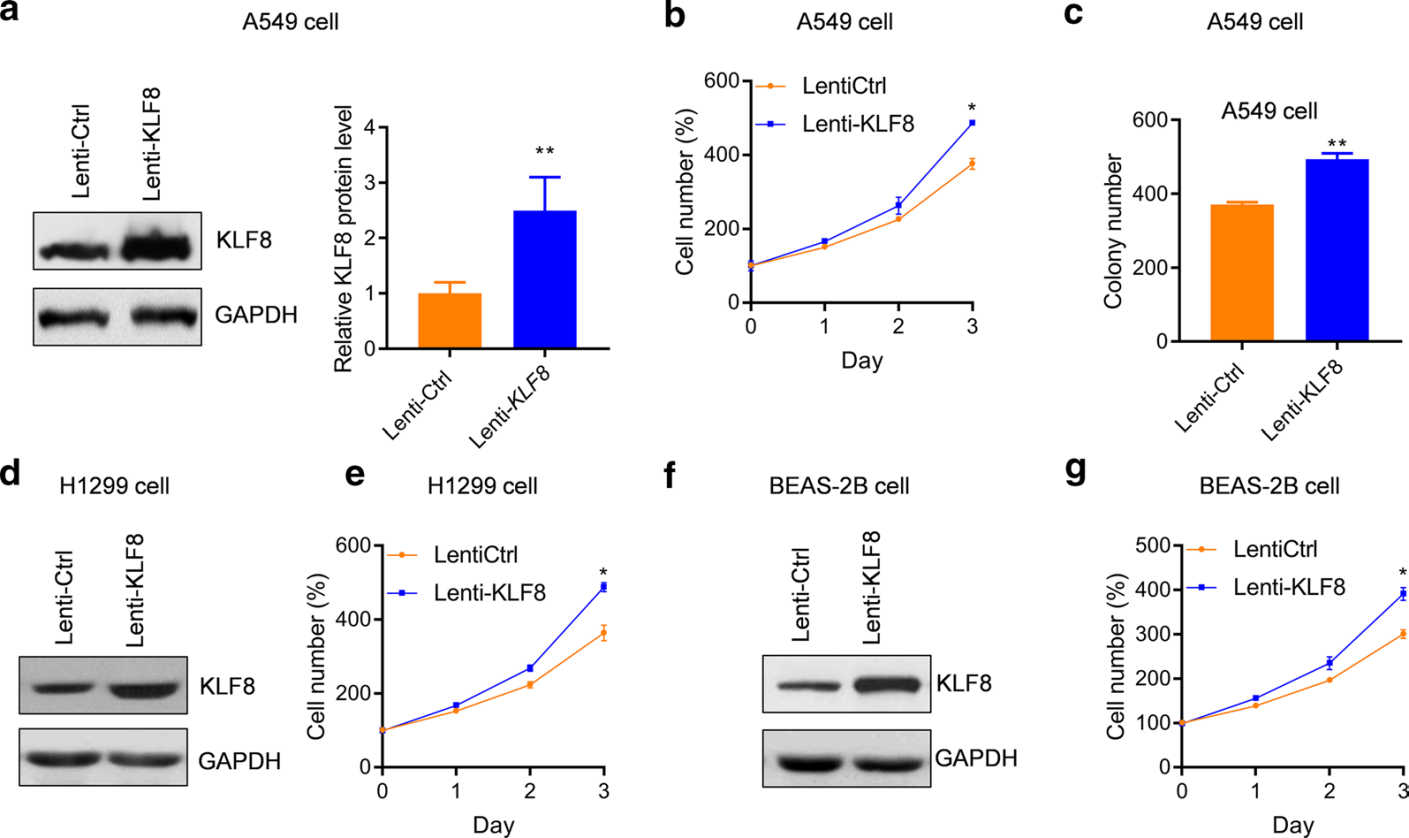
KLF8 overexpression promotes the growth of lung cancer cells in vitro. **a** A549 cells were infected with lentivirus carrying *KLF8* or control constructs and the cells were selected with puromycin. The transduced cells were subjected to analyze the protein level of KLF8. **b**, **c** A549 cells with/without *KLF8* transduction were subjected to proliferation assay (**b**) and colony formation assay (**c**). *p < 0.05 *vs.* Lenti-Ctrl. n = 3 in each group. The number of cells was normalized to the control group at day 0 (%) and the percentage of cell number was shown. **d** H1299 cells were infected with lentivirus carrying *KLF8* or control constructs and the cells were selected with puromycin. **e** H1299 cells with/without *KLF8* transduction were subjected to proliferation assay. *p < 0.05 *vs.* Lenti-Ctrl. n = 3 in each group. **f** BEAS-2B cells were infected with lentivirus carrying *KLF8* or control constructs and the cells were selected with puromycin. **g** BEAS-2B cells with/without *KLF8* transduction were subjected to proliferation. *p < 0.05 *vs.* Lenti-Ctrl. n = 3 in each group

To validate the roles of *KLF8* in human lung cancer, the expressions of *KLF8* in lung cancer cell lines A549 and H1299 were knocked down with lentivirus-mediated shRNA. qRT-PCR and western blot analysis demonstrated that *KLF8* was significantly knocked down by lentivirus-mediated shRNA in these two lung cancer cell lines (Fig. 2a–d). We next prepared A549 and H1299 cells with stable *KLF8* knockdown and those cells were subjected to analyze the effects of *KLF8* on lung cancer cell growth. The cellular proliferation assay showed that the proliferation rate of A549 cells was markedly reduced by *KLF8* knockdown since day 3 (Fig. 2e). The further colony formation assay also demonstrated that *KLF8* knockdown decreased the colony formation capacity of A549 cells (Fig. 2f). Similar effects of *KLF8* knockdown on cell proliferation and colony formation were observed in H1299 cells (Fig. 2g, h). To explore the effects of *KLF8* high expression on lung cancer cells, we generated A549 cells with *KLF8* stable overexpression by infecting the cells with lentivirus-mediated overexpression of KLF8 and selecting with puromycin (Fig. 3a). The cell proliferation and colony formation assay demonstrated that A549 cell growth was significantly enhanced by *KLF8* overexpression (Fig. 3b, c). The promoting effect of KLF8 on cell proliferation was also observed in H1299 lung cancer cells and BEAS-2B non-cancerous bronchial epithelial cells (Fig. 3d–g). These results demonstrated that KLF8 promotes the growth of lung cancer cells.

### KLF8 promotes cell cycle but not affect cell apoptosis of lung cancer cells

**Fig. 4:**
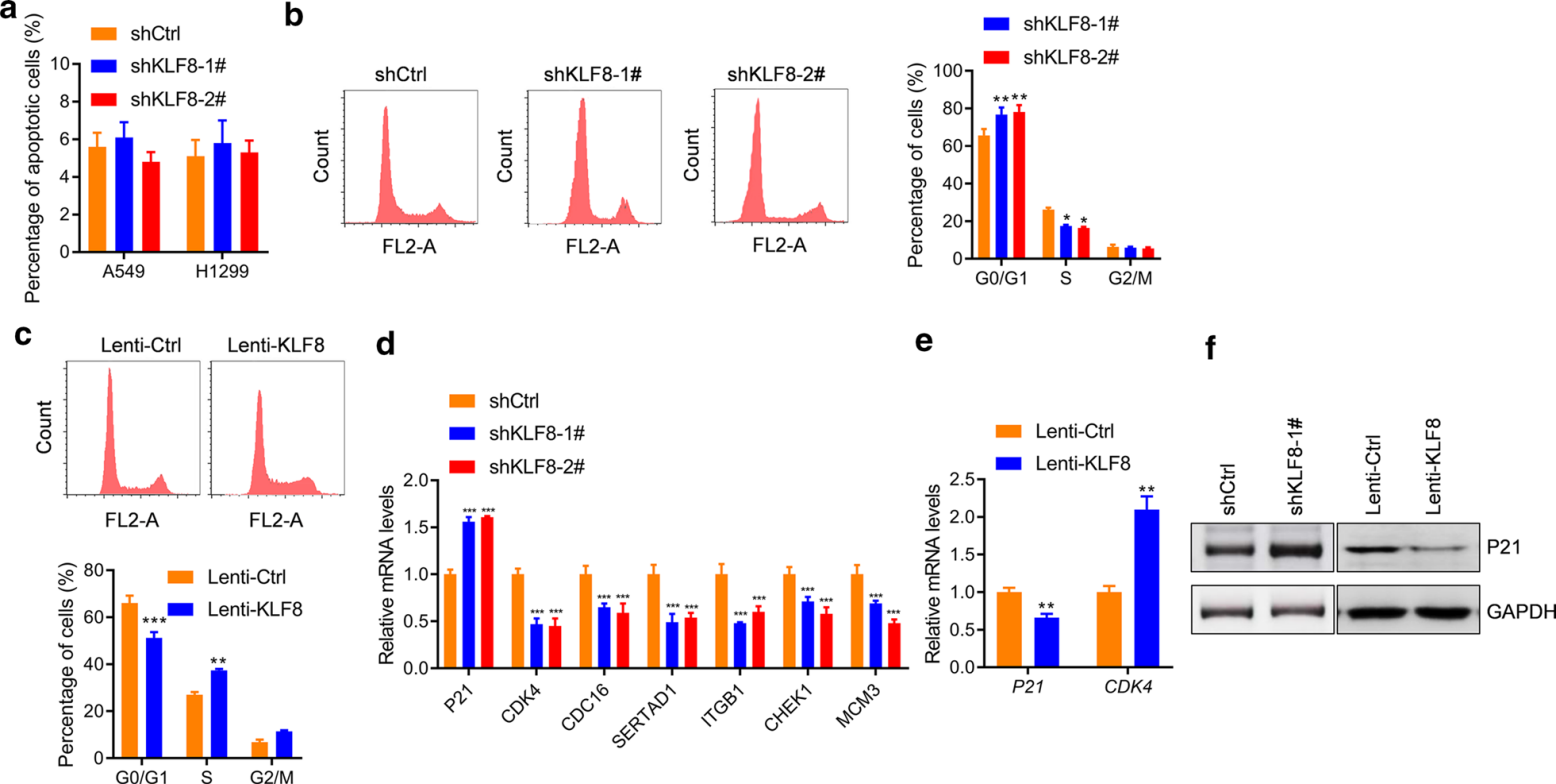
KLF8 regulates cell cycle. **a** KLF8 effects on cellular apoptosis. A549 cells were infected with indicated lentivirus for 24 h, then cellular apoptosis was analyzed. n = 3 in each group. **b** *KLF8* knockdown effects on cell cycle. A549 cells were infected with indicated lentivirus for 24 h, then the cell cycle was analyzed. ** *p *< 0.01 *vs.* shCtrl. n = 3 in each group. **c** *KLF8* overexpression effects on cell cycle. A549 cells were infected with indicated lentivirus for 24 h, then the cell cycle was analyzed. **p < 0.01 and ***p < 0.001 *vs.* shCtrl. n = 3 in each group. **d** Effects of *KLF8* knockdown on the expression of cell cycle regulators. A549 cells were infected with indicated lentivirus for 24 h, then the mRNA level of cell cycle regulators was analyzed. ***p < 0.001 *vs.* shCtrl. n = 3 in each group. **e** Effects of *KLF8* overexpression on the expression of cell cycle regulators. A549 cells were infected with indicated lentivirus for 24 h, then the mRNA level of cell cycle regulators was analyzed. **p < 0.01 *vs.* Ctrl. n = 3 in each group. **f** Western blot analysis of the effects of KLF8 on P21 expression in A549 cells. The cells were treated as in **d** and **e**

We next explored the mechanism by which KLF8 contributes to the hyper-growth of lung cancer cells. We first analyzed the effects of KLF8 on cell survival of lung cancer cells since apoptosis-resistance is a key feature of lung cancer cells. The results showed that *KLF8* knockdown did not induce apoptosis of A549 cells (Fig. 4a and Additional file 1: Figure S1). We next analyzed the effects of *KLF8* on cell cycle of A549 cells. The results showed that *KLF8* knockdown induced a rest of cell cycle at G0/G1 phase whereas *KLF8* overexpression promoted the entry of S phase (Fig. 4b, c). Therefore, *KLF8* controlled cell cycle but not survival of lung cancer cells. We also analyzed the effects of *KLF8* on the expression of cell cycle regulators. The qRT-PCR results showed that *KLF8* knockdown promoted the expression of the cell cycle repressor *P21* whereas reducing the expression of cell cycle promoters, such as *CDK4, CDC16, SERTAD1, ITGB1, CHEK1* and *MCM3* (Fig. 4d). By contrast, *KLF8* overexpression reduced the expression of *P21* and promoted the expression of *CDK4* (Fig. 4e). The western blot result also confirmed the inhibitory effect of KLF8 on P21 expression (Fig. 4f). Taken together, these findings demonstrated that KLF8 regulated the cell cycle of lung cancer cells.

### JMJD2A is involved in the function of KFL8

**Fig. 5:**
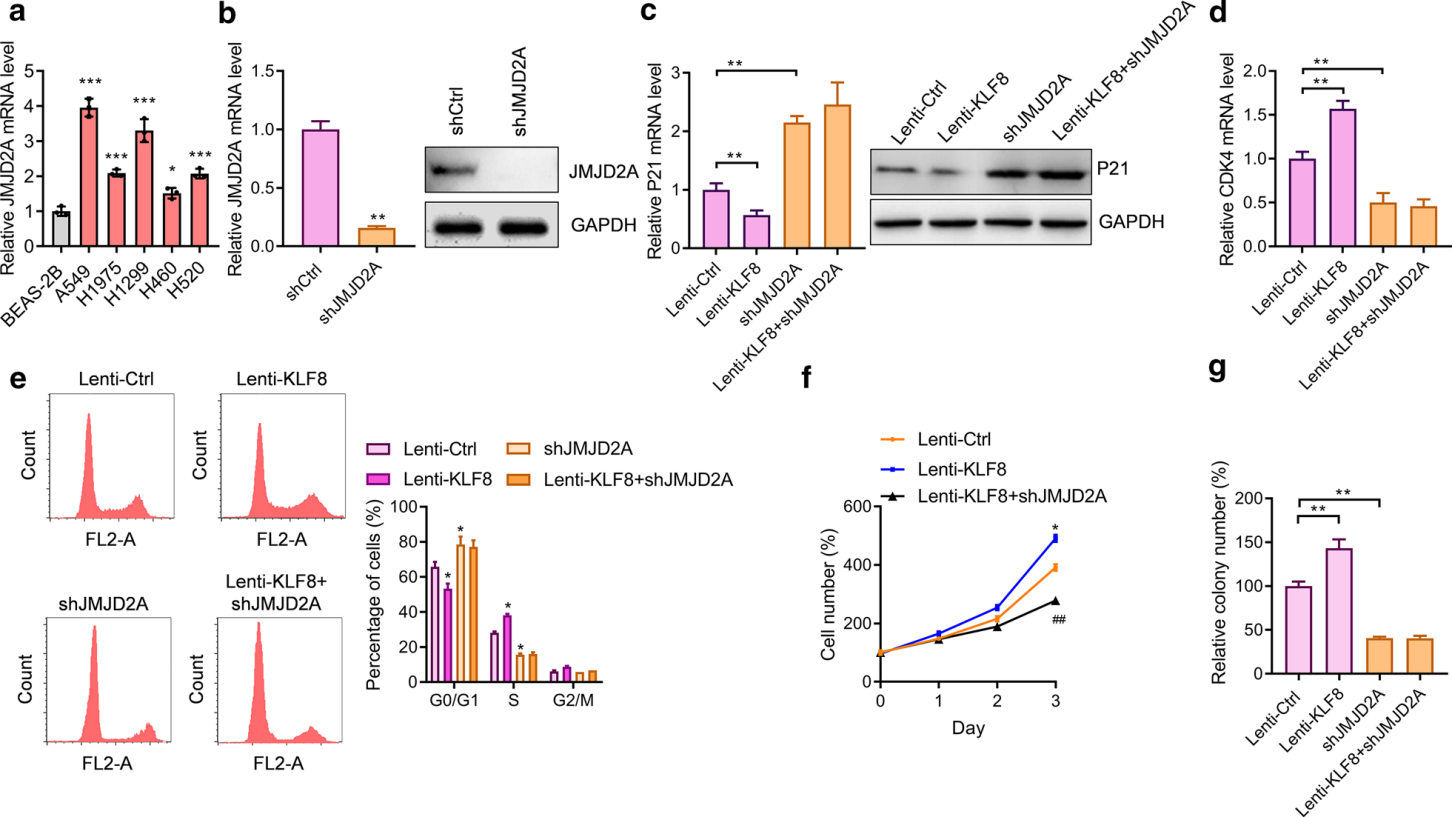
JMJD2A is involved in the regulation of the cell cycle and cell growth by KLF8. **a** The mRNA level of *JMJD2A* is overexpressed in human lung cancer cell lines (A549, H1975, H1299, H460, and H520) compared with normal lung epithelial cell line (BEAS-2B). *p < 0.05 and ***p < 0.001. n = 3 in each group. **b** *JMJD2A* knockdown in A549 cells. A549 cells were infected with indicated lentivirus for 24 h, then the mRNA and protein levels of *JMJD2A* were analyzed. *p < 0.05 *vs.* shCtrl. n = 3 in each group. **c**, **d** JMJD2A knockdown blocks KLF8 effects on the expression of *P21* and *CDK4*. A549 cells were infected with indicated lentivirus for 24 h, then the mRNA and protein level of *P21* (**c**) and *CDK4* (**d**) were analyzed. **p < 0.01. n = 3 in each group. **e** *JMJD2A* knockdown blocks KLF8 effects on cell cycle. A549 cells were infected with indicated lentivirus for 24 h, then the cell cycle was analyzed. *p < 0.05 *vs.* Ctrl. **f**, **g** A549 cells were infected with indicated lentivirus and selected with puromycin and then the transduced cells were subjected to cell proliferation (**f**) and colony formation assay (**g**). *p < 0.05 *vs.* Ctrl. ^##^*p *< 0.01 *vs.* Lenti-*KLF8*. n = 3 in each group. The number of cells was normalized to the control group at day 0 (%) and the percentage of cell number was shown

JMJD2A is a histone demethylase that was reported to regulate lung cancer cell [14]. JMJD2A was highly expressed in human lung cancer cells A549 and H1299 (Fig. 5a). We investigated whether JMJD2A was involved in the function of KLF8 in lung cancer cells. To this end, we knocked down the expression of *JMJD2A* with lentivirus-mediated shRNA. qRT-PCR and western blot assay showed that JMJD2A was significantly reduced by lentivirus-mediated sh*JMJD2A* in A549 cells (Fig. 5b). Similar to *KLF8* knockdown, *JMJD2A* knockdown promoted the expression of *P21* and repressed the expression of *CDK4*. Significantly, *JMJD2A* knockdown blocked the effects of *KLF8* overexpression on the expression of *P21* and *CDK4* in A549 cells (Fig. 5c, d). In addition, we analyzed the effects of *JMJD2A* on cell cycle and found that *JMJD2A* knockdown promoted the entry of S phase and blocked the effects of *KLF8* (Fig. 5e). Finally, we performed cell proliferation and colony formation assay and observed that *JMJD2A* knockdown blocked the effects of *KLF8* overexpression on cell proliferation and colony formation in A549 cells (Fig. 5f, g). Taken together, these findings showed that JMJD2A contributed to the function of KLF8 in human lung cancer cells.

### KLF8 promotes the expression of JMJD2A

**Fig. 6:**
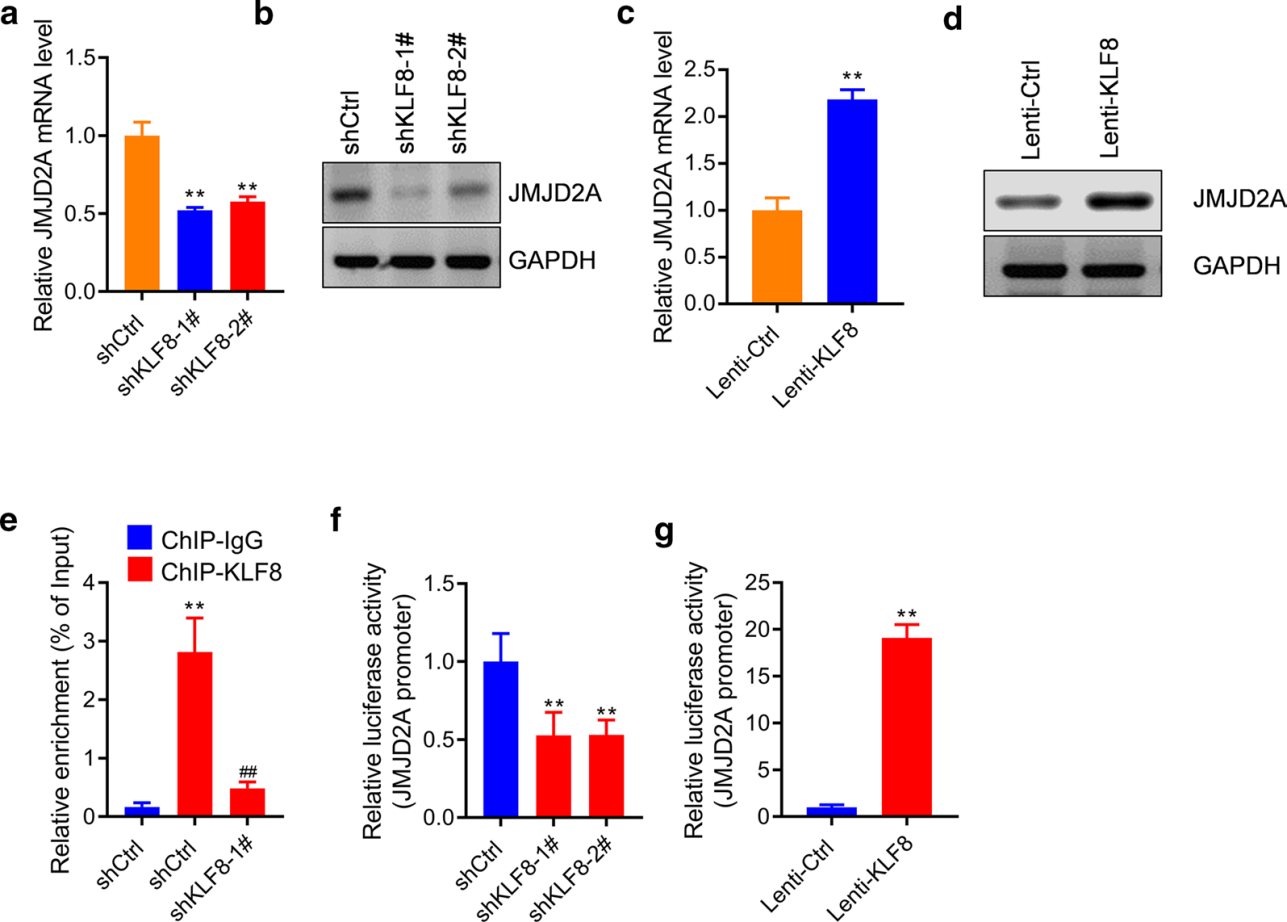
KLF8 promotes the expression of JMJD2A. **a**, **b** *KLF8* knockdown reduces the expression of JMJD2A. A549 cells were infected with indicated lentivirus for 24 h and then the mRNA (**a**) and protein (**b**) level of JMJD2A were analyzed. ** *p *< 0.01 *vs.* shCtrl. n = 3 in each group. **c**, **d** *KLF8* overexpression promotes the expression of *JMJD2A*. A549 cells were infected with indicated lentivirus for 24 h and then the mRNA (**a**) and protein (**b**) level of *JMJD2A* were analyzed. n = 3 in each group. **e** Chromatin-immunoprecipitation (ChIP) assay showing KLF8 binding to *JMJD2A* promoter. **p < 0.01 *vs.* shCtrl + ChIP-IgG, ^##^*p *< 0.01 *vs.* shCtrl + ChIP-*KLF8*. n = 3 in each group. **f** *KLF8* knockdown reduces the promoter activity of *JMJD2A*. A549 cells with/without sh*KLF8* transduction were transfected with pGL3-*JMJD2A* for 24 h, then cells were subjected to luciferase analysis. **p < 0.01 *vs.* shCtrl. n = 3 in each group. **g** *KLF8* overexpression increases the promoter activity of JMJD2A. A549 cells with/without KLF8 transduction were transfected with pGL3-JMJD2A for 24 h, then cells were subjected to luciferase analysis. **p < 0.01 *vs*. Lenti-Ctrl. n = 3 in each group

Since JMJD2A was involved in the function of KLF8 in lung cancer cells, we next analyzed whether KLF8 could regulate the expression of *JMJD2A*. Our qRT-PCR and western blot results showed that *KLF8* knockdown reduced the expression of *JMJD2A* whereas *KLF8* overexpression promoted the expression of *JMJD2A* in A549 cells (Fig. 6a–d). The bioinformatic analysis revealed that there are four KLF8 binding motifs (CACCC) at the promoter of JMJD2A (Additional file 1: Figure S2). We next analyzed whether KLF8 bound the promoter of *JMJD2A*. To this end, we performed chromatin immunoprecipitation (ChIP) assay and observed that KLF8 could bound to *JMJD2A* promoter, which was reduced by *KLF8* knockdown (Fig. 6e). We next cloned the promoter of *JMJD2A* and performed luciferase assay to determine the effects of KLF8 on *JMJD2A* promoter activity in A549 cells. The results showed that *KLF8* knockdown reduced the promoter activity of *JMJD2A* whereas *KLF8* overexpression promoted the promoter activity of *JMJD2A* (Fig. 6f, g). Taken together, these findings demonstrated that KLF8 promotes the expression of JMJD2A by binding its promoter.

## Discussion

In the present work, we provided evidence that the transcriptional factor KLF8 functions as a favorable factor for the growth of human lung cancer cells. We observed that the expression level of *KLF8* was remarkedly upregulated in human lung cancer tissues. Further *gain*-*of*-*function* and *loss*-*of*-*function* experiments demonstrated that KLF8 facilitated the growth of lung cancer cells A549 and H1299. KLF8 regulated the cell cycle but not apoptosis of lung cancer cells depending upon the histone demethylase JMJD2A. Mechanism study revealed that KLF8 bound the promoter of JMJD2A and promoted the expression of JMJD2A.

The roles of KLF8 in cancer biology was widely investigated. KLF8 transcriptionally participates in oncogenic transformation [6]. KLF8 transcription is activated by FAK in human ovarian cancer cells [19]. In breast cancer, KLF8 promotes cell invasion and metastasis by activating the expression of MMP9 in breast cancer cells [7]. In human hepatocellular carcinoma, up-regulation of KLF8 promotes tumor invasion and indicates poor prognosis [20]. KLF8 overexpression is correlated with angiogenesis and poor prognosis in gastric cancer [21]. However, the roles of KLF8 in human lung cancer remain largely unknown. Here we observed that the expression levels of KLF8 were significantly overexpressed in human lung cancer tissues compared with non-cancer tissues. When our manuscript was under preparation, another two reports also reported that *KLF8* mRNA and protein levels were overexpression in human lung cancer tissues and the high expression of KLF8 was significantly correlated with TNM stage, lymph node metastasis and poor overall survive [22, 23]. Therefore, KLF8 may serve as a potential prognostic factor for predicting the progress and outcome of patients with lung cancer.

By utilizing lentivirus-mediated knockdown and overexpression of *KLF8*, we demonstrated that KLF8 promoted the growth (proliferation and colony formation) of lung cancer cells A549 and H1299. Indeed, KLF8 promoted the growth of almost all types of cancer cells, including breast cancer [7], gastric cancer [21], hepatocellular carcinoma [20], ovarian cancer [19], and bladder cancer [24]. In glioma cells, KLF8 also promotes temozolomide resistance by activating β-catenin [25]. In some types of cancer cells, KLF8 promoted the transformation and metastasis of the cancer cells [6, 7]. For instance, KLF8 favors breast cancer cell invasion and metastasis by promoting the expression of MMP9 [7]. In breast cancer cells, KLF8 also cooperated with FAK to enrich the active MMP14 on the cell surface to facilitate the metastasis of the cancer cells [26]. High expression of KLF8 was correlated with TNM stage, lymph node metastasis and poor overall survive [22, 23]. Therefore, it is interesting to explore whether KLF8 regulates the transformation and metastasis of lung cancer cells in further work.

We also found that KLF8 regulated the cell cycle but not survival of lung cancer cells. Knockdown of *KLF8* induced a cell cycle arrest at the G1 phase. KLF8 promoted the expression of the cell cycle regulator *CDK4* and repressed the cell cycle inhibitor *P21*, implicating that KLF8 might regulate the expression of these genes in an indirect manner. Indeed, we observed KLF8 regulated the expression of *CDK4* and *P21* depending upon JMJD2A, which also contributed to the functions of KLF8 in regulating cell cycle and growth of lung cancer cells.

JMJD2A was also reported to be overexpressed and regulate the cell cycle in diverse types of cancer, including human lung cancer [12–16]. However, the mechanism underlying JMJD2A up-regulation in human lung cancer remains unknown. Here we provided evidence for one of the potential mechanisms that contributed to the upregulation of JMJD2A in human lung cancer cells. We identified several KLF8-binding motifs at the promoter of JMJD2A and our ChIP data and luciferase results revealed that KLF8 bound the promoter of JMJD2A and promoted the promoter activity of JMJD2A and subsequently the expression of JMJD2A.

## Conclusions

In conclusion, we demonstrated that KLF8-JMJD2A signaling controls the cell cycle of human lung cancer cells. Thus KLF8 may serve as a potential target for the treatment of human lung cancer.

## Supplementary information

**Additional file 1.** Additional data and figures.

## Data Availability

The analyzed data sets generated during the study are available from the corresponding author on reasonable request.

## Abbreviations

KLF8: kruppel like factor 8
FAK: focal adhesion kinase
MMP9: matrix metalloproteinase 9
