# Supplementary material for: KLF8 overexpression promotes the growth of human lung cancer cells by promoting the expression of JMJD2A
Source: Cancer Cell Int. 2019 Oct 7;19:258. doi: 10.1186/s12935-019-0970-3 (PMC6781403; doi:10.1186/s12935-019-0970-3)
Supplement: Supplementary file 1 — Additional file 1. Additional data and figures. [file 12935_2019_970_MOESM1_ESM.docx]

**Additional Data**


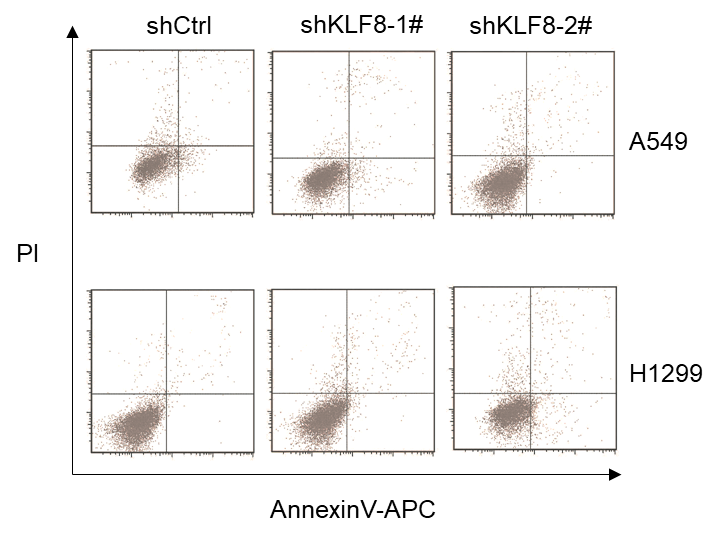


**Additional Figure S1 Representative data of flow analysis of apoptotic cells (Related to Figure 4A).**


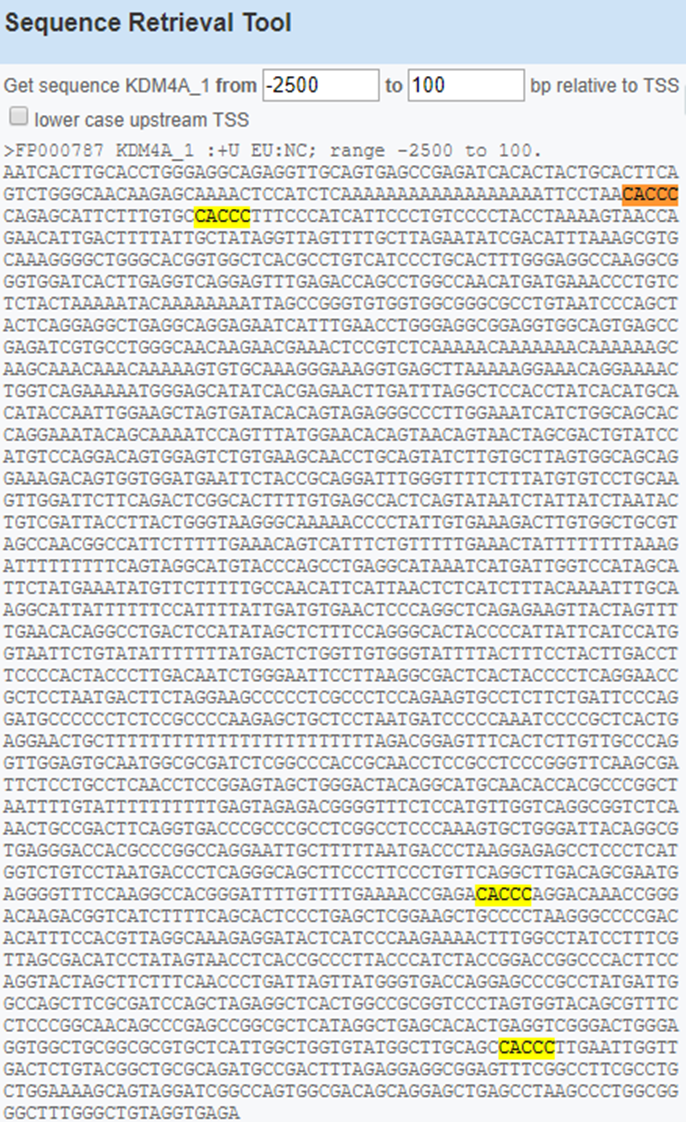


**Additional Figure S2 Binding motifs (CACCC) of KLF8 at the promoter of JMJD2A (-2500bp to +100 bp).**
